# Assessment of the efficacy of using taurine supplements to improve growth and feed utilization of juvenile starry flounder (*Platichthys stellatus*) given diets based on soy-protein

**DOI:** 10.7717/peerj.10597

**Published:** 2021-01-11

**Authors:** Peiyu Li, Hongyi Bu, Baoshan Li, Yongzhi Sun, Meiqi Wang, Zhidong Song

**Affiliations:** 1 Shandong Marine Resource and Environment Research Institute, Yantai, China; 2 Rizhao Polytechnic, Rizhao, China; 3 Shanghai Ocean University, Shanghai, China

**Keywords:** Feed additives, Digestive enzymes, Hepatic enzymes, Antioxidant biomarkers, Body composition

## Abstract

A feeding trial was conducted to assess the feasibility of supplementing taurine in soy-based diets for juvenile starry flounder *Platichthys stellatus*. The basal diet (Crude protein 66.5%, crude lipid 8.5%) was supplemented with 0 (control), 0.5%, 1.0%, 1.5%, 2.0% and 2.5% taurine to formulate six test diets. Each diet was fed to 40 juvenile fish (22.25 g) in triplicate tanks (120 L) attached to a sea water circulation-system. Fish were fed twice daily by hand to apparent satiation during the 56-d trial. At the end of the trial, fish were counted and weighed for the analyses of growth performance, diet utilization and survival after a 24-h fast. Blood, intestines and muscles were collected for the analyses of serum oxidation resistance, digestive enzymes and body compostion. Livers were collected from the remaining fish at 4 h post-feeding for metabolic enzymes analyses. The results showed that fish fed diets supplemented with 1.0–2.5% taurine grew from 22.25–22.26 g to 47.88–50.40 g with higher average weight gain (25.62–28.12 vs 23.07 g ), specific growth rate (1.37–1.46 vs 1.27%/d ), feed intake (1.04–1.06 vs 1.00%/d), protein efficiency (2.50–2.61 vs 2.44) and lower feed conversion rate (0.84–0.83 vs 0.89) than the control treatment. Diets supplemented with 1.5–2.5% taurine significantly elevated the activities of pepsin (2.47–2.55 vs 2.22, U mg^−1^ prot), trypsin of distal intestine(14.55–15.24 vs 11.94, U mg^−1^ prot), hepatic glucokinase (126.62–129.42 vs 105.56, U mg^−1^ prot) and fatty acid synthetase (125.56-136.89 vs 108.45, U mg^−1^ prot). All diets supplemented with taurine increased the activities of lipase (32.23–36.67 vs 29.53, U g^−1^ prot) and trypsin (35.85–37.89 vs 33.54, U mg^−1^ prot) of proximal intestine, hepatic aspartate transaminase (736.990–832.38 vs 699.24, U mg^−1^ prot), alanine aminotransferase (477.40–551.86 vs 373.97, U mg^−1^ prot) and glycogen synthase (2.16–2.59 vs 1.97, U mg^−1^ prot), as well as serum superoxide dismutase (4.33–4.59 vs 4.07, U mg^−1^ prot ) and glutathione peroxidase (42.23–50.25 vs 39.17, mol mg^−1^ prot). Therefore, taurine supplementation benefits juvenile starry flounder growth, digestion, nutrients metabolism and oxidation resistance. The optimal taurine requirement for starry flounder is 1.75%, and the recommended supplementation level is at least 1.6% for maximizing growth of fish fed a low-fishmeal diet (13.6%).

## Introduction

In the past few decades, plant-based protein sources have been receiving considerable attention as a partial or total fishmeal replacer in the aquafeed industry (Tacon et al., 2011). However, removing fishmeal from the diet by incorporating plant protein reduces the content of several important bioactive substance especially taurine. Taurine is found in relatively high concentration in fishmeal but is almost nonexistent in most plant protein sources. In fish, taurine is involved in a particularly wide variety of functions including minerals absorption, bile acid conjugation, osmoregulation, antioxidation and modulation of immunity. Higher plant-based diet intake is associated with taurine deficiency, of which symptoms include reduced growth and survival, increased susceptibility to diseases, and impaired larval development (reviewed by Salze & Davis, 2015).

Several studies reveal that the ability to synthesize endogenous taurine varies greatly among fish species due to differences in the activity of key enzymes on the pathway of taurine biosynthesis, and taurine biosynthesis seem to be high in several freshwater fish such as rainbow trout *Oncorhynchus mykiss* and common carp *Cyprinus carpio* but low in most marine species such as Japanese flounder *Paralichthys olivaceus* and turbot *Scophthalmus maximus* (Goto et al., 2001; Goto et al., 2003; Yokoyama & Nakazoe, 1992; Yokoyama et al., 2001; Park et al., 2002; Kim et al., 2008; Wang et al., 2014). As a result, low biosynthesis ability makes taurine an limiting nutrient for these marine species once these fish fed plant-based diets (Jirsa et al., 2014). Increasing evidences indicate dietary taurine supplementation has more often been shown to give positive effects in several marine species including turbot, Janpanese flounder, rock bream *Oplegnathus fasciatus*, seabass *Atractoscion nobilis*, sea bream *Pagrus major*, yellowtail *Seriola lalandi* and Florida pompano *Trachinotus carolinus* with results suggesting that the optimal taurine levels in diets of these fish range widely from 0.25% to 1.56% depending on fish species and growth stages (Qi et al., 2012; Park et al., 2002; Ferreira et al., 2014; Martins et al., 2018; Matsunari et al., 2008; Salze & Davis, 2014; Salze et al., 2018).

Starry flounder *Platichthys stellatus* is an economically valuable coastal resident fish species. Annual global catch of this fish has suffered major declines in past years (An et al., 2014). With the establishment of a mass production technique for seedling starry flounder recently, the starry flounder farming is booming in Korea and China (Tian et al., 2016). Nutrition and feeding play important roles in supporting the rapid development of this fish species aquaculture. A few of studies have assessed the nutrients requirements of this species such as protein (Lee, Kim & Cho, 2006), essential fatty acids (Lee, Lee & Kim, 2003) and protein to lipid ratio (Wang et al., 2017). A recent study that evaluated tolerance of starry flounder to soy protein, suggested that dietary maximum fishmeal replacement level with soy protein concentrate was 40% (Li et al., 2015). In that study, taurine deficiency in diet was supposed to be one of the key factors for poor growth performance of fish fed soy-based diets. Therefore, the present study was conducted to investigate the compensating effect of supplemental taurine in soy-based diets for starry flounder on impaired growth performance, digestion and metabolism, to help understand the role of taurine in fish when dietary fishmeal is replaced by plant protein sources in a high level.

## Materials and Methods

### Experimental diets

A plant protein-based diet formula containing 13.6% fishmeal (Crude protein 66.5%; crude lipid 8.5%; American Seafoods Company, Seattle, WA, USA) and 55.8% soy protein concentrate (Crude protein 67.0%; crude lipid 0.8%; Shandong Changrun biology Co.Ltd, Linyi, China) was employed in this study as a basal diet formula (Table 1). This formula had been proven to significantly retard fish growth compared to the fishmeal-based diet in our previous study (Li et al., 2015). Taurine (purity, 99.5%; Hongsheng Biological Technology Co., Ltd, Suzhou, China) was added to the basal diet at 0.5%(T0.5), 1.0% (T1.0), 1.5% (T1.5), 2.0% (T2.0) and 2.5% (T2.5) to give 6 test diets in total, including the basal diet (control, T0) (Table 1). Experimental diets were prepared by mixing the dry components thoroughly in a Patterson-Kelley twin shell^®^ Batch V-mixer (Patterson-Kelley Co.Inc., East Stroudsburg, PA), followed by the addition of fish oil (RongchengLitai fishmeal Co.Ltd, Rongcheng, China) and distilled water. The mixed dough was passed through a screw pelleting machine to make moist pellets (3 mm diameter). After air-drying to moisture content below 8%, all diets were frozen at −20 °C until use. The measured taurine levels are 0.11%, 0.57%, 1.08%, 1.69%, 2.10% and 2.58%, respectively.

**Table 1:** Formula and proximate composition of the experimental diets (% dry basis).

Ingredients	Experimental diets					
	T0	T0.5	T1.0	T1.5	T2.0	T2.5
Fishmeal	13.6	13.6	13.6	13.6	13.6	13.6
Soy protein concentrate	55.8	55.8	55.8	55.8	55.8	55.8
Fish oil	8.6	8.6	8.6	8.6	8.6	8.6
Soybean lecithin	1.0	1.0	1.0	1.0	1.0	1.0
*α*-starch	6.44	6.44	6.44	6.44	6.44	6.44
Mineral mixture ^a^	2.0	2.0	2.0	2.0	2.0	2.0
Vitamin mixture ^b^	1.0	1.0	1.0	1.0	1.0	1.0
Carboxymethyl cellulose	9.6	9.1	8.6	8.1	7.6	7.1
lysine	0.5	0.5	0.5	0.5	0.5	0.5
methionine	0.4	0.4	0.4	0.4	0.4	0.4
Sulfo-betaine	0.5	0.5	0.5	0.5	0.5	0.5
Choline chloride	0.5	0.5	0.5	0.5	0.5	0.5
Antioxidant	0.06	0.06	0.06	0.06	0.06	0.06
Taurine	0.0	0.5	1.0	1.5	2.0	2.5
Total	100.0	100.0	100.0	100.0	100.0	100.0
Proximate composition (Measured value, % dry material)						
Crude protein	50.14	50.42	50.69	50.95	51.25	51.54
Crude lipid	11.71	11.73	11.68	11.73	11.70	11.74
Ash	10.40	10.45	10.39	10.42	10.42	10.44
Taurine	0.11	0.57	1.08	1.69	2.10	2.58
Gross energy (kJ g^−1^)	20.64	20.65	20.68	20.60	20.62	20.63

**Notes.**

a Mineral mixture (mg kg^−1^ diet): MgSO_4_⋅ 7H_2_O, 3568.0; NaH_2_PO_4_⋅ 2H_2_O, 25568.0; KCl, 3020.5; KAl (SO_4_)_2_, 8.3; CoCl_2_, 28.0; ZnSO_4_⋅ 7H_2_O, 353.0; Ca-lactate, 15968.0; CuSO_4_⋅ 5H_2_O, 9.0; KI, 7.0; MnSO_4_⋅ 4H_2_O, 63.1; Na_2_SeO_3_, 1.5; C_6_H_5_O_7_Fe⋅ 5H_2_O,1533.0; NaCl,100.0; NaF,4.0.

b Vitamin mixture (mg kg^−1^ diet): Retinol acetate, 38.0; Cholecalciferol, 13.2; Alpha-tocopherol, 210.0; Thiamin, 115.0; Riboflavin, 380.0; Pyridoxine HCl, 88.0; Pantothenic acid, 368.0; Niacin acid, 1030.0; Biotin, 10.0; Folic acid, 20.0; Vitamin B_12_, 1.3; Inositol, 4000.0; Ascorbic acid, 500.0; Vitamin K, 10.0.

### Experimental protocol

Juvenile starry flounder was purchased from a commercial fish nursery (Rongcheng, China). The feeding trial was performed in an indoor recirculating aquaculture system (RAS) of Shandong Marine Resource and Environment Research Institute (Yantai, China). An ultraviolet disinfection system consisted of four UV lamps (230w) and duct in which the sea water flowed. Synthetic fiber filter cotton (updated once a month) and coral sand populated with nitrifying/denitrifying bacteria were used to remove wastes and keep ammonia-N concentration below 0.05 mg L^−1^. A fixed photoperiod, controlled by timers and fluorescent lights, was followed (14 h daylight: 10 h dark). Water temperature was maintained at 17.0 °C by air conditioner. Dissolved oxygen was maintained above 7.7 mg L^−1^ by air pump (500w). Water turnover rate was maintained at 50% by adjusting water pump flow. Sodium carbonate was added every three days to keep pH value constant (8.1). Salinity was maintained at 33‰  by adding freshwater every week. These water quality parameters were monitored every 30 min.

All fish were reared in RAS and fed the control diet (T0) for two weeks, in order to acclimatize the experimental conditions before the trial began. When the trial began, all fish were individually weighed and 40 fish averaging initial wet body weight of 22.25 g were randomly distributed in group to the 18 tanks (120-L). Fish of triplicate tanks were fed one of the test diets at a 3% body weight/day during the 8-week trial. The dead fish were weighed and recorded daily for calculation of survival rate.

### Sampling protocol

At the end of the feeding trial, all fish were fasted for 24 h. Fish in each tank were counted and weighed for the analysis of growth performance, diet utilization and survival rate. Twelve fish per tank were randomly selected and immersed in MS-222 (3-Aminobenzoic acid ethyl ester methanesulfonate) solution (200 mg L^−1^) for 20 min, until loss of voluntary movement was observed. Individual weight and length were measured, and then blood was immediately collected by caudal puncture using heparinized syringes (ammonium salt heparin, 1000 U/ml, Sigma-Aldrich). Plasma was separated by centrifugation at 12,000 g for 20 min at 4 °C and stored at −80 °C for analysis of oxidation resistance. Nine of the sampled fish were killed by beating the head and dissected to collect dorsal muscle, entire viscera, liver and intestine. The entire viscera, liver and intestine were weighed for calculation of organ indices. The livers and dorsal muscles were collected for chemical composition analysis. The remaining fish were refed for 7 more days. Six fish were sampled from each tank at 4 h after the morning meal and livers were collected for postprandial metabolic enzymes analysis. The samples of intestines and livers were prepared according to a modified method reported previously by Castro et al. (2018). In brief, the samples were homogenated in 4 vol. of ice-cold 100 mM Tris–HCl buffer (pH 7.8) containing 0.1 mM EDTA and 0.1% (v/v) Triton X-100. Homogenates were centrifuged at 6,000 g for 20 min at 4 °C and then the supernatants were kept in aliquots and stored at −80  °C for analysis.

The feeding trial management and sampling were approved by Shandong Marine Resource and Environment Research Institute Aquatic Experimental Animal Ethics Committee (No. 201946). All measures were made to reduce the stress and pain of experimental animals.

### Sample analyses

#### Calculation of growth performance, diet utilization and organ coefficients

The variables of growth performance, diet utilization and organ coefficients were calculated as follows:

Average weight gain (AWG, g) = W_t_ (g) –W_i_ (g)

Specific growth rate (SGR, %/d) = (lnW_t_–lnW_i_) /t ×100

Daily feed intake (FI, %/d) = feed intake (g)/[(W_t_+ W_i_)/ 2 ×t] ×100

Feed conversion ratio (FCR) =dry feed intake (g)/wet weight gain(g)

Protein efficiency ratio (PER) = weight gain (g)/ingested protein (g)

Survival rate (SR, %) = final fish number/initial fish number ×100

Condition factor (CF, %) = body weight (g)/body length(cm)^3^ ×100

Viscerasomatic index (VSI, %) = visceral weight (g)/body weight (g) ×100

Hepatosomatic index (HSI, %) = hepatic weight(g)/body weight (g) ×100

where W_i_ and W_t_ were average initial weight (g) and average final weight (g); t was the experimental duration (d).

#### Nutrition composition of diet, liver and muscle

Diets, livers and dorsal muscles were dried to constant weight at 105 °C in an oven for determination of water content. After grinding, these samples were analyzed for the content of crude protein, crude lipid, crude ash and enery. Protein (N × 6.25) was determined by using the Kjeldahl digestion method, crude lipid by using the Soxhlet extraction method, and crude ash by combustion at 550 °C (AOAC, 2000). Energy was determined using an adiabatic bomb calorimeter (IKA^®^C 6000, Janke& Kunkel KG.IKA-werk, German). Amino acid composition was determined using an amino acid analyzer (Hitachi L8900, Hitachi High-Technologies Corporation, Japan). In brief, muscle sample was hydrolyzed with 6 ml of 6N HCl at 110  °C for 22 h in an evacuated sealed tube. The hydrolysate was dried with nitrogen gas to remove HCl and redissolved in 0.1N HCl loading buffer. After filtration through 0.22-µm polyethersulphone ultrafiltration membrane, the hydrolysate was analyzed by amino acid analyzer.

#### Activity analysis of digestive enzyme

The activities of digestive enzymes were determined at 37  °C and the changes in absorbance were monitored using a spectrophotometer (TU-1810DSPC, Beijing Persee General Instrument Co., Ltd, Beijing). To normalize enzyme activity, total protein content of the supernatant was determined by the Bradford assay (1976) using bovine serum albumin as a standard.

The specific assay condition for each enzyme was as follows:

The activity of pepsin was analyzed using hemoglobin as the substrate. 0.5 ml of enzyme dilution was added to 2.5 ml preheated (37 °C) hemoglobin substrate (1g hemoglobin was dissolved in 100 ml 65 mM HCl solution, pH 2.0) and incubated at 37 °C for exactly 10 min. The reaction was stopped by adding 5 ml of 5% TCA and removed from bath after 5 min and filtered. The absorbance at 280 nm of filtrate was recorded. One activity unit (U mg^−1^prot) of pepsin is defined as the amount of enzyme of 1 mg tissue homogenate that hydrolyzed hemoglobin to form 1 µg tyrosine equivalent per minute at 37 °C, pH 2.0.

The activity of trypsin was measured with N *α*-Benzoyl-L-arginine ethyl ester (BAEE) as substrate. BAEE was dissolved in buffer (67 mM sodium phosphate buffer, pH 7.6, 25 °C) at a final concentration of 0.25 mM. Intestinal extract (50 µL) were added to 1.5ml substrate solution at 37 °C, and the change of absorbance at 253 nm was recorded for 20 min. One activity unit (U mg^−1^prot) of trypsin is defined as the amount of enzyme of 1 mg tissue homogenate that produce a ΔA253 of 0.003 per minute with BAEE as substrate at pH 7.6 at 37 °C in a reaction volume of 1.55 ml.

The activities of lipase and amylase were analyzed using commercial kits purchased from Nanjing Jiancheng Bioengineering Institute (Nanjing, China) following the manufacturer’s instructions. One unit (U mg^−1^ prot) of lipase is defined as the amount of enzyme of 1 mg tissue homogenate that hydrolyzes 1 µmol of triglyceride per minute at 37  °C, pH 9.0. One unit (U mg^−1^ prot) of amylase is defined as the amount of enzyme of 1 mg tissue homogenate that liberates 1.0 mg of maltose from starch in 3 min at 20 °C, pH 6.9.

#### Activity analysis of metabolic enzyme

The liver homogenate was used for activity analysis of metabolic enzyme. The activities of aspartate transaminase (AST) and alanine transaminase (ALT) were measured by automatic biochemistry analyzer (Hitachi 7020, Tokyo, Japan). One unit (U g^−1^prot) of AST is defined as the amount of enzyme in 1g tissue protein that generated 1.0 µmol of glutamate per minute at pH 8.0 and 37 °C. One unit (U g^−1^prot) of ALT is defined as the amount of enzyme in 1g tissue protein that generated 1.0µmol of pyruvate per minute, at pH8.0 and 37 °C. Fatty acid synthase (FAS), hepatic lipase (HL), glycogen synthetase (GCS), glucokinase (GK), pyruvate carboxylase (PC) were analyzed using Activity Assay Kit (Solarbio Science & Technology Co., Ltd, Beijing, PR China). One unit (U mg^−1^ prot) of FAS activity was defined as the amount of enzyme in 1 mg tissue protein that oxidize 1.0 µmol NADPH per minute at pH 7.8 and 37 °C. One unit (U mg^−1^prot) of HL activity is defined as the amount of enzyme in 1 mg tissue protein that generate 1.0 µmol naphthylester from *α*-naphthalene acetate per minute at pH 7.8 and 37 °C. One unit (U mg^−1^prot) of GCS activity is defined as the amount of enzyme in 1 mg tissue protein that oxidize 1.0 nmol NADH per minute at pH 7.8 and 37 °C. One unit (U mg^−1^prot) of GK activity is defined as the amount of enzyme in 1 mg tissue protein convert 1 pmol of NADP to NADPH at 30 °C. One unit (U mg^−1^prot) of PC activity is defined as the amount of enzyme in 1 mg tissue protein convert 1.0 mmol of pyruvate and CO_2_ to oxalacetate per minute at pH 7.8 at 30  °C.

Activity of hepatic cysteine sulfinate decarboxylase (CSD) was determined according to the method reported by Goto et al. (2001), Goto et al. (2003) and Yokoyama et al. (2001). In brief, isolated livers were homogenized in 0.25 M sucrose and dialyzed against 10 mM phosphate buffer (pH 7.4) for 4 h to remove the endogenous taurine at 4 °C.The incubation mixture consisted of 100 mM sodium phosphate buffer (pH 7.2), 1.0 mM cysteinesulfinate, 0.2 mM pyridoxal 5′-phosphate, 4 mM 2-mercaptoethanol. The reaction was started by the addition of the crude enzyme solution (1 mg protein) and the incubation was continued at 35 °C for 1 h. After the reaction mixture was heated at 70  °C for 3 min to end the reaction, *β*-alanine (0.2 mmol) was added as an internal standard. Amino acid analysis was performed by reverse-phase HPLC with o-phthalaldehyde as a prelabeling. The taurine content formed by the H_2_O_2_ treatment was calculated by the area ratio of taurine to *β*-alanine and regarded as the enzyme activity.

#### Serum oxidation resistance

The activities of serum superoxide dismutase (SOD), catalase (CAT) and glutathione peroxidase (GSH-Px) as well as the malonyldialdehyde (MDA) level were measured by the method of Noori, Nasir & Mahboob (2009).

### Statistical analysis

The software SPSS, 11.0 microcomputer software package (SPSS, Chicago, IL, USA) was used for all statistical evaluations. A homogeneity test for the variance was conducted. All data were subjected to one-way analysis of variance (ANOVA) followed by Tukey’s test. Differences were considered as significant when *P* < 0.05. Data were expressed as mean with Standard Deviation (SD), Pooled S.E, *p*-value and *F*-value.

## Results

### Growth performance, diet utilization and organ indices

**Figure 1:**
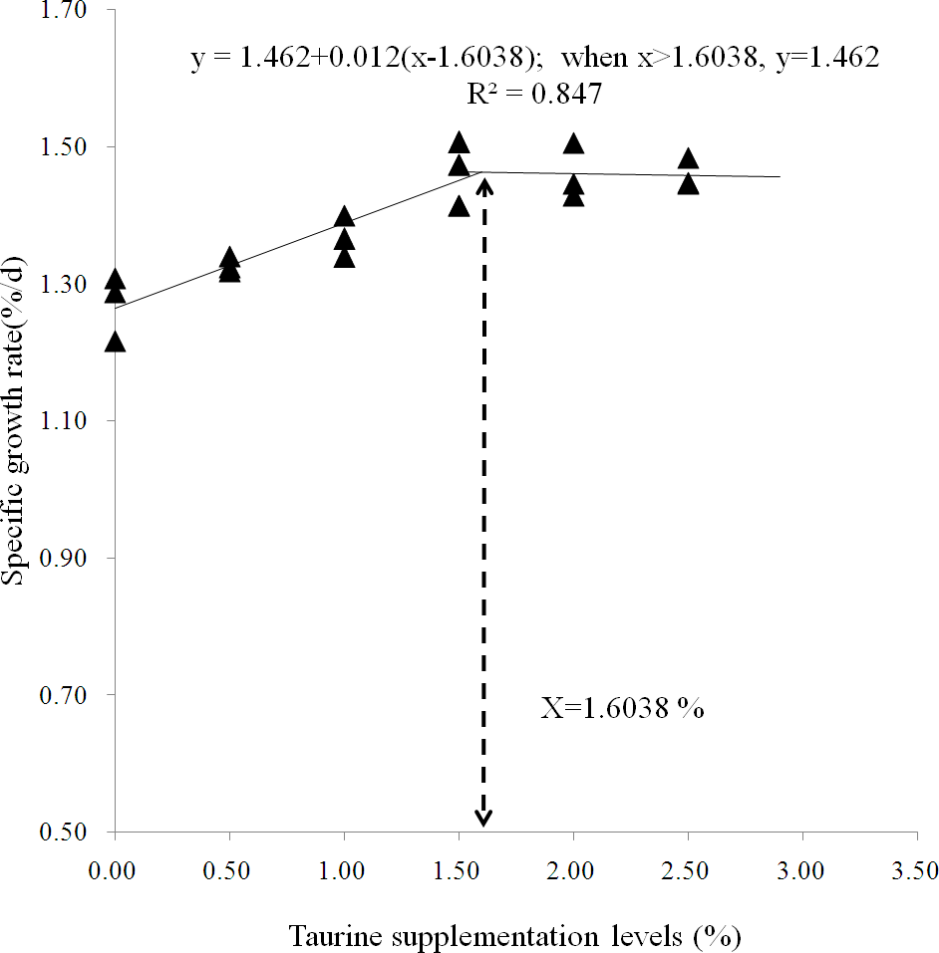
Broken-line model of specific growth rate. Broken-line analysis of specific growth rate ( *Y*-axis, *n* = 3) in juvenile starry flounder *Platichthys stellatus* fed diets supplemented with the different taurine levels for 8 weeks. Values on the *X*-axis are the taurine supplemention levels in experimental diets. The broken line regression analysis indicates that the optimal taurine supplementation level is 1.60% for growth (correspondingly, the minimum taurine requirement is 1.75 %).

As summarized in Table 2, SR was above 90% and not significantly affected by dietary treatments (*P* > 0.05). AWG, SGR and PER gradually increased until the taurine supplementation level increased up to 1.5%, and then reached a plateau. Diets supplemented with ≥1% taurine significantly improved AWG, SGR and FI, while diets supplemented with ≥1.5% taurine significantly increased PER, VSI and HSI and reduced FCR as compared with the control treatment (*P* < 0.05). All diets supplemented with taurine significantly increased CF (*P* < 0.05). A broken-line model predicted that the optimal taurine requirement for starry flounder is 1.75% and the recommended supplementation level is at least 1.6% for maximizing fish growth (Fig. 1).

**Table 2:** Growth performance, diet utilization and organ indices of fish fed diets supplemented with graded levels of taurine.

	Experimental diets						Pooled S.E	*p*-value	*F*-value
	T0	T0.5	T1.0	T1.5	T2.0	T2.5			
**Growth performance**									
Initial weight (g)	22.24 ± 0.02	22.23 ± 0.05	22.26 ± 0.03	22.25 ± 0.02	22.25 ± 0.03	22.25 ± 0.03	0.006	0.869	0.356
Final weight (g)	45.31 ± 1.23 ^a^	46.75 ± 0.22 ^ab^	47.88 ± 0.92 ^b^	50.34 ± 1.33 ^c^	50.40 ± 1.21 ^c^	50.37 ± 0.67 ^c^	0.518	0.000	17.511
AWG (g)^1^	23.07 ± 1.22 ^a^	24.51 ± 0.26 ^ab^	25.62 ± 0.81 ^b^	28.29 ± 1.33 ^c^	28.15 ± 1.18 ^c^	28.12 ± 0.64 ^c^	0.530	0.000	15.466
SGR (%/d)^2^	1.27 ± 0.05 ^a^	1.33 ± 0.01 ^ab^	1.37 ± 0.03 ^b^	1.46 ± 0.05 ^b^	1.46 ± 0.04 ^b^	1.46 ± 0.02 ^b^	0.020	0.000	17.039
SR (%)^3^	100 ± 0.00	100 ± 0.00	100 ± 0.16	99.07 ± 0.00	100 ± 0.00	100 ± 0.00	0.154	0.458	1.000
**Diet utilization**									
FI^4^	1.00 ± 0.02 ^a^	1.02 ± 0.02 ^ab^	1.04 ± 0.01 ^b^	1.06 ± 0.01 ^c^	1.04 ± 0.01 ^bc^	1.04 ± 0.01 ^bc^	0.006	0.003	6.860
FCR^5^	0.87 ± 0.03 ^b^	0.86 ± 0.01 ^ab^	0.84 ± 0.01 ^ab^	0.84 ± 0.03 ^a^	0.83 ± 0.01 ^a^	0.83 ± 0.01 ^a^	0.006	0.027	3.817
PER^6^	2.44 ± 0.09 ^a^	2.49 ± 0.04 ^ab^	2.53 ± 0.16 ^ab^	2.59 ± 0.09 ^b^	2.61 ± 0.05 ^b^	2.61 ± 0.02 ^b^	0.023	0.145	2.038
Organ coefficients									
CF^7^	2.52 ± 0.04 ^a^	2.75 ± 0.08 ^b^	3.00 ± 0.09 ^c^	3.03 ± 0.07 ^c^	3.01 ± 0.13 ^c^	3.06 ± 0.07 ^c^	0.051	0.000	20.103
VSI^8^	4.14 ± 0.15 ^ab^	4.28 ± 0.11 ^a^	4.22 ± 0.14 ^ab^	4.56 ± 0.18 ^c^	4.52 ± 0.11 ^c^	4.44 ± 0.06 ^bc^	0.046	0.008	5.391
HSI^9^	0.92 ± 0.06 ^a^	1.11 ± 0.13 ^ab^	1.08 ± 0.13 ^ab^	1.26 ± 0.17 ^b^	1.21 ± 0.15 ^b^	1.23 ± 0.08 ^b^	0.038	0.053	3.053

**Notes.**

Values (Means ±S.D.) ( *n* = 3) in the same row with different superscripts show significant difference (*P* <0.05).

1 AWG: Average weight gain (g).

2 SGR: Specific growth rate (%/d).

3 SR: Survival rate (%).

4 FI: Daily feeding intake (%/d).

5 FCR: Feed conversion ratio.

6 PER: Protein efficiency ratio.

7 CF: Condition factor (%).

8 VSI: Viscerasomatic index (%).

9 HSI: Hepatosomatic index (%).

### Nutritional composition of liver and dorsal muscle

As summarized in Table 3, the moisture and fat contents of muscle as well as the ash content of liver were not altered by the taurine supplementation (*P* > 0.05). All diets supplemented with taurine significantly reduced moisture content of liver but increased protein content of liver as well as the taurine contents of liver and muscle (*P* <  0.05). Diets supplemented with ≥1% taurine significantly increased fat content of liver and cysteine content of muscle while reduced ash content of muscle (*P* <  0.05). Diets supplemented with ≥2% taurine significantly increased protein, methionine and phenylalanine contents of muscle (*P* <  0.05).

**Table 3:** Nutritional composition of different tissues in fish fed diets supplemented with graded levels of taurine.

	Experimental diets							Pooled SE	*p*-value	*F*-value
		T0	T0.5	T1.0	T1.5	T2.0	T2.5			
**Liver (% wet weight)**										
Moisture		66.63 ± 0.47^c^	65.49 ± 0.26^b^	65.34 ± 0.48^b^	64.86 ± 0.44^ab^	64.80 ± 0.30^ab^	64.21 ± 0.35^a^	0.201	0.000	11.469
Crude protein		12.45 ± 0.10^a^	12.71 ± 0.18^b^	12.75 ± 0.09^bc^	12.91 ± 0.09^bc^	12.85 ± 0.11^bc^	12.97 ± 0.04^c^	0.047	0.002	7.959
Crude fat		16.50 ± 0.34^a^	16.98 ± 0.21^ab^	17.25 ± 0.39^bc^	17.43 ± 0.25^bc^	17.64 ± 0.14^c^	17.72 ± 0.32^c^	0.119	0.004	6.342
Ash		1.49 ± 0.07	1.43 ± 0.04	1.39 ± 0.14	1.42 ± 0.15	1.42 ± 0.07	1.41 ± 0.04	0.020	0.829	0.415
Taurine (mg 100g^−1^ dry weight)		183.16 ± 4.69^a^	371.92 ± 14.59^b^	441.15 ± 9.64^c^	454.81 ± 14.95^c^	448.56 ± 8.12^c^	453.42 ± 3.14^c^	23.798	0.000	329.251
**Muscle ( % wet weight)**										
Moisture	74.12 ± 0.35		74.27 ± 0.34	74.46 ± 0.16	74.50 ± 0.35	74.58 ± 0.28	74.45 ± 0.12	0.068	0.429	1.058
Crude protein	23.27 ± 0.23^a^		23.29 ± 0.19^a^	23.40 ± 0.13^ab^	23.52 ± 0.18^ab^	23.66 ± 0.07^b^	23.68 ± 0.28^b^	0.055	0.080	2.618
Crude fat	1.78 ± 0.14		1.77 ± 0.15	1.82 ± 0.08	1.88 ± 0.12	1.80 ± 0.07	1.85 ± 0.11	0.026	0.846	0.390
Ash	0.44 ± 0.00^c^		0.44 ± 0.00^bc^	0.43 ± 0.08^ab^	0.43 ± 0.13^ab^	0.43 ± 0.07^ab^	0.43 ± 0.11^a^	0.005	0.000	13.3
Taurine(mg 100 g^−1^ dry weight)	184.43 ± 13.24^a^		381.20 ± 3.76^b^	769.13 ± 12.81^c^	784.50 ± 6.27^c^	775.81 ± 5.26^c^	777.27 ± 9.48^c^	58,181	0.000	3351.501
**Muscle amino acid composition ( % dry weight)**										
Aspartic acid		9.76 ± 0.64	9.79 ± 0.33	9.63 ± 0.54	9.31 ± 0.15	9.71 ± 0.07	9.64 ± 0.16	0.084	0.673	0.641
Serine		4.23 ± 0.26	4.21 ± 0.12	4.18 ± 0.30	3.98 ± 0.06	4.26 ± 0.11	4.13 ± 0.14	0.043	0.546	0.840
Glutamic acid		16.20 ± 1.08	16.31 ± 0.57	15.86 ± 0.89	15.45 ± 0.28	16.13 ± 0.27	16.21 ± 0.26	0.147	0.604	0.746
Glycine		5.66 ± 0.36	5.47 ± 0.15	5.35 ± 0.61	5.22 ± 0.18	5.55 ± 0.10	5.44 ± 0.15	0.071	0.618	0.724
Alanine		6.54 ± 0.44	6.57 ± 0.23	6.47 ± 0.46	6.29 ± 0.11	6.56 ± 0.13	6.60 ± 0.18	0.063	0.790	0.472
Cysteine		1.18 ± 0.13^a^	1.27 ± 0.08^ab^	1.23 ± 0.01^b^	1.38 ± 0.09^b^	1.35 ± 0.09^b^	1.37 ± 0.08^b^	0.025	0.082	2.597
Tyrosine		3.10 ± 0.19	3.12 ± 0.22	3.18 ± 0.10	3.02 ± 0.15	3.20 ± 0.09	3.03 ± 0.16	0.035	0.629	0.707
Proline		2.84 ± 0.16	2.74 ± 0.08	2.65 ± 0.22	2.58 ± 0.06	2.75 ± 0.08	2.65 ±0.14	0.034	0.288	1.412
Threonine		4.29 ± 0.27	4.30 ± 0.14	4.22 ± 0.25	4.07 ± 0.06	4.25 ± 0.03	4.17 ± 0.13	0.039	0.597	0.758
Valine		4.49 ± 0.30	4.53 ± 0.13	4.40 ± 0.24	4.29 ± 0.05	4.45 ± 0.09	4.53 ± 0.09	0.040	0.571	0.800
Methionine		1.68 ± 0.07^a^	1.76 ± 0.09^ab^	1.79 ± 0.04^ab^	1.86 ± 0.08^ab^	1.96 ± 0.20^b^	1.92 ± 0.10^b^	0.031	0.067	2.802
Isoleucine		4.17 ± 0.29	4.22 ± 0.25	4.24 ± 0.24	4.11 ± 0.08	4.27 ± 0.04	4.28 ± 0.11	0.041	0.886	0.328
Leucine		7.11 ± 0.48	7.26 ± 0.40	7.29 ± 0.40	7.08 ± 0.17	7.33 ± 0.13	7.31 ± 0.10	0.068	0.877	0.343
Phenylalanine		3.24 ± 0.21^a^	3.39 ± 0.32^ab^	3.52 ± 0.22^ab^	3.50 ± 0.18^ab^	3.76 ± 0.11^b^	3.74 ± 0.23^b^	0.064	0.107	2.326
Lysine		7.40 ± 0.48	7.56 ± 0.49	7.72 ± 0.38	7.43 ± 0.24	7.70 ± 0.26	7.42 ± 0.11	0.078	0.780	0.487
Histidine		2.14 ± 0.10	2.18 ± 0.17	2.33 ± 0.14	2.18 ± 0.05	2.30 ± 0.10	2.29 ± 0.13	0.030	0.321	1.315
Arginine		5.89 ± 0.36	5.88 ± 0.15	5.79 ± 0.39	5.64 ± 0.11	5.95 ± 0.14	5.76 ± 0.35	0.060	0.773	0.497

### Digestive enzyme activity

As presented in Table 4, the amylase activity of proximal intestine (PI), the lipase and amylase activities of distal intestine (DI) were not affected by dietary treatments (*P* > 0.05). All diets supplemented with taurine significantly elevated the activities of lipase and trypsin of PI (*P* < 0.05). Diets supplemented with ≥ 1.5% taurine significantly increased pepsin activity of stomach and trypsin activity of DI (*P* < 0.05).

**Table 4:** Digestive enzyme activity of fish fed diets supplemented with graded levels of taurine.

	Experimental diets						Pooled SE	*p*-value	F-value
	T0	T0.5	T1.0	T1.5	T2.0	T2.5			
**Stomach**										
Pepsin (U mg^−1^ prot)	2.22 ± 0.09^a^	2.19 ± 0.12^a^	2.32 ± 0.10^ab^	2.55 ± 0.14^c^	2.51 ± 0.07^bc^	2.47 ± 0.06^bc^	0.039	0.003	6.921
**Proximal intestine**										
Trypsin (U mg^−1^ prot)	33.54 ± 0.59^a^	35.85 ± 0.91^b^	36.76 ± 0.77^bc^	37.89 ± 1.01^c^	37.50 ± 0.49^c^	36.85 ± 0.80^bc^	0.368	0.000	11.150
Lipase (U g^−1^ prot)	29.53 ± 1.75^a^	32.23 ± 1.07^b^	34.42 ± 0.98^c^	35.68 ± 0.83^cd^	36.40 ± 0.83^cd^	36.67 ± 0.92^d^	0.655	0.000	18.973
Amylase (U g^−1^ prot)	6.33 ± 0.10	6.36 ± 0.08	6.36 ± 0.15	6.39 ± 0.17	6.35 ± 0.14	6.43 ± 0.11	0.027	0.938	0.238
**Distal intestine**		
Trypsin (U mg^−1^ prot)	11.94 ± 0.39 ^a^	11.91 ± 0.63 ^a^	13.24 ± 0.99 ^ab^	14.55 ± 0.54 ^bc^	14.94 ± 1.47 ^bc^	15.24 ± 1.16 ^c^	0.380	0.002	7.440
Lipase (U g^−1^ prot)	10.80 ± 1.42	10.27 ± 0.66	10.11 ± 0.77	10.12 ± 0.65	9.73 ± 0.53	10.88 ± 0.67	0.185	0.682	0.628
Amylase (U g^−1^ prot)	2.08 ± 0.17	2.10 ± 0.16	2.22 ± 0.24	2.20 ± 0.16	2.00 ± 0.18	2.05 ± 0.15	0.041	0.619	0.722

**Notes.**

Values (Means ± S.D.) (*n* = 3) in the same row with different superscripts show significant difference (*P* < 0.05).

### Hepatic metabolic enzyme activity

As presented in Table 5, all diets supplemented with taurine significantly increased activities of hepatic AST, ALT and GCS (*P* < 0.05). Diets supplemented with ≥1% taurine increased FAS activity (*P* < 0.05) and diets with ≥1.5% taurine increased GK activity (*P* < 0.05). The hepatic CSD activity was lower in fish fed diets with 2.0% and 2.5% taurine than that of the control treatment (*P* < 0.05). No difference was found on HL activity in all treatments (*P* > 0.05). The PC activity was higher in fish fed 1.0% taurine-supplemented diet than in fish fed 2.0% and 2.5% taurine-supplemented diets (*P* < 0.05), but was similar to that of fish fed other diets (*P* > 0.05).

**Table 5:** The 4-hour postprandial hepatic metabolic enzyme activity of fish fed diets supplemented with graded levels of taurine.

	Experimental diets						Pooled SE	*p*-value	*F*-value
	T0	T0.5	T1.0	T1.5	T2.0	T2.5			
AST (U mg^−1^ prot)^1^	699.24 ± 18.17^a^	736.99 ± 8.20^b^	814.29 ± 9.07^c^	826.93 ± 9.23^c^	830.83 ± 8.82^c^	832.38 ± 6.04^c^	12.878	0.000	87.277
ALT (U mg^−1^ prot)^2^	373.97 ± 14.34^a^	477.40 ± 7.46^b^	506.77 ± 21.96^c^	534.59 ± 4.78^d^	543.96 ± 8.41^d^	551.86 ± 7.30^d^	14.968	0.000	86.710
FAS (U mg^−1^ prot)^3^	108.45 ± 7.12^a^	117.36 ± 5.58^ab^	138.37 ± 6.93^c^	136.89 ± 5.72^c^	125.56 ± 9.28^bc^	127.73 ± 7.55^bc^	2.868	0.001	8.673
HL (U mg^−1^ prot)^4^	133.80 ± 4.73	128.59 ± 19.42	131.92 ± 4.88	131.54 ± 9.69	130.41 ± 7.66	136.91 ± 9.80	2.191	0.946	0.222
GCS (U mg^−1^ prot)^5^	1.97 ± 0.50^a^	2.19 ± 0.18^bc^	2.59 ± 0.19^c^	2.22 ± 0.19^bc^	2.18 ± 0.15^bc^	2.16 ± 0.18^bc^	0.068	0.175	1.865
GK (U mg^−1^ prot)^6^	105.56 ± 13.98^a^	97.71 ± 12.93^a^	111.55 ± 13.08^ab^	129.42 ± 4.59^b^	126.62 ± 7.30^b^	127.95 ± 8.15^b^	3.635	0.012	4.791
PC (U mg^−1^ prot)^7^	127.93 ± 6.78^abc^	136.43 ± 9.33^bc^	138.20 ± 6.37^c^	136.14 ± 3.82^bc^	124.86 ± 4.90^ab^	121.56 ± 2.81^a^	1.957	0.023	3.987
CSD (nmol min^−1^mg protein ^−1^)^8^	0.25 ± 0.02 ^c^	0.23 ± 0.02 ^bc^	0.23 ± 0.03 ^bc^	0.22 ± 0.03 ^abc^	0.21 ± 0.01 ^ab^	0.19 ± 0.01 ^a^	0.012	0.033	3.558

**Notes.**

Values (Means ±S.D.) ( *n* = 3) in the same row with different superscripts show significant difference (*P* <0.05).

1 AST: aspartate transaminase.

2 ALT: alanine aminotransferase.

3 FAS: fatty acid synthetase.

4 HL: hepatic lipase.

5 GCS: glycogen synthase.

6 GK: glucokinase.

7 PC: pyruvate carboxylase.

8 CSD: cysteine sulfinate decarboxylase.

### Serum antioxidant enzyme activity

As presented in Table 6, serum CAT activity and MDA content were not affected by dietary treatments (*P* > 0.05), but the activities of SOD and GSH-Px were elevated in all taurine-supplemented groups compared with that of the control group (*P* < 0.05).

**Table 6:** Serum oxidation resistance of fish fed diets supplemented with graded levels of taurine.

	Experimental diets						Pooled SE	*p*-value	*F-value*
	T0	T0.5	T1.0	T1.5	T2.0	T2.5			
SOD (10^3^ U L^−1^)^1^	4.07 ± 0.20^a^	4.33 ± 0.09^b^	4.54 ± 0.07^b^, ^c^	4.57 ± 0.10^c^	4.59 ± 0.14^c^	4.59 ± 0.09^c^	0.052	0.006	8.785
GSH-Px (U L^−1^)^2^	39.17 ± 1.99^c^	42.38 ± 1.36^b^	42.23 ± 1.42^b^	44.21 ± 1.15^b^	51.11 ± 1.82^c^	50.25 ± 1.64^c^	1.103	0.000	26.931
CAT (U mg^−1^)^3^	181.90 ± 6.72	178.57 ± 7.36	183.28 ± 8.38	178.09 ± 10.72	173.80 ± 14.14	193.67 ± 8.57	2.394	0.301	1.372
MDA (mol L^−1^)^4^	1.08 ± 0.21	1.02 ± 0.15	0.97 ± 0.09	0.86 ± 0.11	0.85 ± 0.11	0.83 ± 0.12	0.035	0.194	1.770

**Notes.**

Values (Means ±S.D.) ( *n* = 3) in the same row with different superscripts show significant difference (*P* <0.05).

1 SOD: Superoxide dismutase

2 GSH-Px: Glutathione peroxidase

3 CAT: Catalase

4 MDA: Malonyldialdehyde

## Discussion

The results from the present study demonstrated low to moderate (0.5–1.5%) taurine supplementation improved juvenile fish growth in a dose-dependent manner. This positive growth response implicated that the endogenous synthesis of taurine was inadequate for optimal growth in starry flounder and juvenile fish could utilize efficiently exogenous taurine. Similar findings were also reported in other marine fish, such as yellowtail (Tochino et al., 2009), Florida pompano. (Rossi, 2011), seabass *Dicentrarchus labrax* (Martinez et al., 2004; Kotzamanis et al., 2012; Rimoldi et al., 2016; Martins et al., 2018; Ceccotti et al., 2019), juvenile cobia *Rachycentron canadum* (Lunger et al., 2007), Japanese flounder (Park et al., 2002; Kim et al., 2005) and turbot (Wang et al., 2014). However, It was noted that the growth did not continue to increase while reached a pleatue value though the taurine supplementation level further increased. The dietary taurine supplementation level that yielded maximum specific growth rate was 1.6% based on a broken-line model estimation of growth performance, predicting that the taurine requirement of juvenile fish was 1.7%. The estimated requirement was higher than those for juvenile rock bream *Oplegnathus fasciatus* (Ferreira et al., 2014), red sea bream *Pagrus major* ((Matsunari et al., 2008) and Nile tilapia *Oreochromis niloticus* (Al-Feky et al., 2016) but was close to the range of 1.2–1.6% for Japanese flounder (Park et al., 2002; Kim et al., 2005), and turbot (Qi et al., 2012).

In the present study, supplementation of 1.0% taurine in diet significantly elevated diet intake FI though the diet utilisation FCR was not significantly altered. According to this phenomenon, the growth benefit of juvenile fish fed low taurine-supplemented diets was due to increased diet consumption but not diet utilization efficiency. Taurine possesses the ability to stimulate feeding in fish, and fish can sense dietary taurine and regulate their feeding activity (Carr, 1982; Carr & Derby, 1986). Therefore, supplementation of taurine improved diet palatability and acceptability which explained the gradually increased feed intake in taurine-supplemented groups. It was interesting that the feed utilization efficiency (FCR and PER) was not significantly improved until the taurine supplementation level reached above 1.5%. According to the digestive enzymes analyses, supplementation of ≥1% taurine both elevated protease (pepsin and trypsin) and lipase activity which were consistent with the findings on larval cobia (Salze, McLean & Craig, 2012) and common dentex *Dentex dentex* (Chatzifotis et al., 2008) fed plant-based diet that supplementation of taurine increased the activities of trypsin and bile salt-activated lipase. These increased digestive enzymes activities explained the better diet utilization in taurine-supplemented group. Taken together, these results demonstrate that there is a threshold effect of taurine supplementation in juvenile starry flounder (in the range of 1–1.5%), in that there is no discernable alteration in diet intake but a clear improvement in diet utilization efficiency.

As juvenile fish develop, the growth of some organs is prioritized and higher than other tissues. This type of growth pattern is called allometric growth (Elin et al., 2016). Information on the allometric growth of different organ groups may contribute to a better understanding of whether different somatic growth rates due to diet quality affect the allometric relative growth rates of internal organs. VSI and HSI are often used as indicators of nutrient storage in viscera and liver. Moreira et al. (2008) reported that an increase in HSI may be related to increased glycogen and lipid deposition in the liver of fish. In the present study, VSI and HSI of fish fed the control diet were much lower than that reported in previous studies (1.92–3.24%, Ding et al., 2009); 2.85–3.0%, Ma et al., 2014). The relatively low VSI and HSI may be related to low nutrients deposition in organs of fish fed plant-based diet, suggesting a state of insufficient reserves of energy and nutrients. Similar phenomena were found on tongue sole *Cynoglossus semilaevis* (Dai et al., 2016) and European sea bass (Geay et al., 2011) when these fish fed plant-based diet. However, diets supplemented with ≥ 1.0% taurine increased VSI and HSI as well as the fat and protein conent of liver (Table 3). This indicate that taurine supplementation contribute to improve diet quality by increasing energy and nutrient reserves in fish organ, which may promote the rapid growth of fish.

A weak activity of hepatic cysteinesulfinate decarboxylase was detected in juvenile starry flounder, which was close to that reported in Japanese flounder and red sea bream by Goto et al. (2001) and Goto et al. (2003), indicating that the ability of these fish species to synthesize taurine was negligible. Therefore, the present result showed that juvenile starry flounder fed the control diet had the lowest taurine accumulation in liver and muscle despite of the highest postprandial hepatic cysteinesulfinate decarboxylase activity. On the other hand, the protein and fat contents of liver as well as the protein content of muscle were lowest in the control group (Table 3). The poor taurine status negatively affected the protein and fat depositon in liver and muscle and thus developed a sign of weak nutrient metabolism and energy metabolism dysfunction as reflected in the lowest activities of metabolic enzymes related to protein, fat and energy metabolism in all treatments (Table 5). However, our results showed that this metabolism dysfunction can be reversed by appropriate taurine supplementation in diet. Although hepatic CSD activity decreased gradually as a negative response to the taurine supplementation level, the taurine contents of liver and muscle were significantly elevated in 1.0–2.5% taurine-supplemented treatments. This indicates that appropriate taurine supplementation (≥1.0%) in diet can make up for the weak synethsis of endogenous taurine and alter the taurine status in tissues.

Hepatic response to nutritional stimuli is characterized by a significant up- and down- regulation on activities of metabolic enzymes involved in the intermediary metabolism. Analyzing activities of hepatic enzymes from a number of metabolic pathways may help to clarify the metabolic alterations caused by taurine supplementation. A number of studies have found that dietary fishmeal replacement with vegetable proteins surpressed several genes expression and metabolic enzymes acitivities, which altered related biological processes including lipid metabolism, protein/amino acid metabolism, carbohydrate metabolism and immune function, as reported in juvenile tilapia *Oreochromis niloticus* × *O. aureus* (Lin & Luo, 2011), Japanese seabass *Lateolabrax japonicus* (Men et al., 2014), juvenile cobia *Rachycentron canadum*, European catfish *Silurus glanis* (Kumar et al., 2017), rainbow trout *Oncorhynchus mykiss* (Panserat et al., 2008), European sea bas*s Dicentrarchus labrax* (Geay et al., 2011). These metabolic alterations were associated with poor growth performance and regarded as metabolic disturbances caused by high vegetable protein intake. In present study, fish fed diets supplemented with taurine upregulated postprandial hepatic ALT and AST activities, which suggested that taurine could improve the capacity of fish hepatocytes to oxidize amino acids or convert amino acids to other intermediates. This result agreed with the findings of Banuelos-Vargas et al. (2014) that taurine restored the activity of key enzymes of hepatic amino acid catabolism in turbot fed soy protein-based diets to levels similar to those fed fishmeal based diet. Consistent with the increased aminotransferase activities, obvious increases in the activities of FAS, GCS and GK in postabsorptive state were detected in the taurine-supplemented groups relative to the control group. These enzymes are key regulatory enzymes in the fatty acid synthesis, glycogenesis and glycolysis. Therefore, The increased activities of these enzymes indicated that taurine enhanced postprandial lipid and energy metabolism of fish hepatocytes, representing a possible recovery from metabolic disturbances.

In the present study, taurine supplementation elevated serum SOD and GSH-Px activities, which were consistent with the published findings that taurine supplementation increased SOD and GSH-Px activities in juvenile black carp *M. piceus* (Zhang et al., 2018), yellow catfish *Pelteobagrus fulvidraco* (Li et al., 2016) and totoaba *Totoaba macdonaldi* (Banuelos-Vargas et al., 2014) fed with plant-based diets. This suggested that taurine could upregulated the synthesis of antioxidant enzymes and improved the oxidation resistence. Several studies on zebrafish (Rosemberg et al., 2010) and European sea bass (Coutinho et al., 2017) reported that taurine could increase expression of anti-oxidative enzymes related genes and thus increase the production of antioxidant enzymes. In addition, the serum MDA contents were constant despite the taurine-mediated elevation of SOD and GSH-Px activities, indicating that the cellular redox homeostasis might be regulated by many factors, not just restricted to taurine.

In conclusion, the physiological effects of taurine are not well defined across fish species. The physiological requirement of taurine may not be met by endogenous synthesis in starry flounder. Appropriate taurine supplementation in a plant protein-based diet can improve growth performance, stimulate feeding, and enhance digestion and metabolism of nutrients. In addition, dietary taurine exerts beneficial effects on antioxidant capacity and nutritional composition of fish. Results from the present study demonstrate that the optimal taurine requirement for starry flounder is 1.7% and the recommended supplementation level is at least 1.6% for maximizing growth of fish fed a low-fishmeal diet (13.6%). This particular area of research is very limited and these results certainly warrant future investigations.

##  Supplemental Information

10.7717/peerj.10597/supp-1 Supplemental Information 1 Taurine improving growth parameters of fish fed low-fishmeal diet as a supplement

## Additional Information and Declarations

### Data Availability

The following information was supplied regarding data availability: Raw data are available as a Supplementary File.
